# Rubisco accumulation factor 1 (Raf1) plays essential roles in mediating Rubisco assembly and carboxysome biogenesis

**DOI:** 10.1073/pnas.2007990117

**Published:** 2020-07-07

**Authors:** Fang Huang, Wen-Wen Kong, Yaqi Sun, Taiyu Chen, Gregory F. Dykes, Yong-Liang Jiang, Lu-Ning Liu

**Affiliations:** ^a^Institute of Integrative Biology, University of Liverpool, L69 7ZB Liverpool, United Kingdom;; ^b^School of Life Sciences, University of Science and Technology of China, Hefei, 230027 Anhui, China;; ^c^College of Marine Life Sciences, Frontiers Science Center for Deep Ocean Multispheres and Earth System, Ocean University of China, 266003 Qingdao, China

**Keywords:** Rubisco accumulation factor 1, Rubisco, carboxysome, carbon fixation, cyanobacteria

## Abstract

Carboxysomes are membrane-free organelles for carbon assimilation in cyanobacteria. The carboxysome consists of a proteinaceous shell that structurally resembles virus capsids and internal enzymes including ribulose 1,5-bisphosphate carboxylase/oxygenase (Rubisco), the primary carbon-fixing enzyme in photosynthesis. The formation of carboxysomes requires hierarchical self-assembly of thousands of protein subunits, initiated from Rubisco assembly and packaging to shell encapsulation. Here we study the role of Rubisco assembly factor 1 (Raf1) in Rubisco assembly and carboxysome formation in a model cyanobacterium, *Synechococcus elongatus* PCC7942 (Syn7942). Cryo-electron microscopy reveals that Raf1 facilitates Rubisco assembly by mediating RbcL dimer formation and dimer–dimer interactions. Syn7942 cells lacking Raf1 are unable to form canonical intact carboxysomes but generate a large number of intermediate assemblies comprising Rubisco, CcaA, CcmM, and CcmN without shell encapsulation and a low abundance of carboxysome-like structures with reduced dimensions and irregular shell shapes and internal organization. As a consequence, the Raf1-depleted cells exhibit reduced Rubisco content, CO_2_-fixing activity, and cell growth. Our results provide mechanistic insight into the chaperone-assisted Rubisco assembly and biogenesis of carboxysomes. Advanced understanding of the biogenesis and stepwise formation process of the biogeochemically important organelle may inform strategies for heterologous engineering of functional CO_2_-fixing modules to improve photosynthesis.

## Significance

Cyanobacteria are keystone organisms in global carbon fixation. Their great carbon-assimilation capability arises from a specialized virus-like protein organelle, the carboxysome, which comprises hundreds of proteins that form a shell to encapsulate the CO_2_-fixing enzymes Rubisco and carbonic anhydrase. How do these proteins self-assemble to construct the defined architecture? Here we explore the significance of one assembly factor, Raf1, in Rubisco assembly and carboxysome formation. We show that Raf1 mediates Rubisco assembly; without Raf1, carboxysome proteins are prone to form intermediate assemblies and small carboxysome-like structures rather than intact carboxysomes. Our results suggest a model of the Raf1-mediated biogenesis of carboxysomes and provide advanced knowledge of carboxysome assembly and function, informing synthetic engineering of functional CO_2_-fixing organelles for biotechnological applications.

Photosynthesis is conceivably the most important biological process on Earth. It comprises light-driven reactions that convert solar energy into chemical energy in the form of NADPH and ATP along with light-independent reactions, known as the Calvin-Benson-Bassham cycle, which fuel sugar production by CO_2_ fixation using the generated NADPH and ATP (1, 2). An ultimate rate-limiting step of photosynthesis is catalyzed by a naturally inefficient enzyme for carbon fixation, ribulose-1,5-bisphosphate carboxylase/oxygenase (Rubisco) (3). The carboxylation activity of Rubisco is remarkably compromised by side reactions with O_2_. How to maximize its carboxylation activity and diminish its oxygenation has been a central engineering goal for improving photosynthetic performance and global agricultural production (4, 5).

Photosynthetic organisms have evolved diverse CO_2_-concentrating mechanisms (CCMs) to elevate the local CO_2_ level in the vicinity of Rubisco and increase Rubisco’s carboxylation efficiency (6, 7). Carboxysomes are a group of bacterial microcompartments that are key for carbon fixation and are the core CCM components in cyanobacteria, which contribute greatly to global carbon assimilation (8). Rubisco enzymes and β-type carbonic anhydrases (CcaAs) are spatially encapsulated and concentrated inside the carboxysome by a virus-like proteinaceous shell (9).

Based on the types of Rubisco encased, carboxysomes can be classified into α- and β-carboxysomes, which are functionally and morphologically similar but assemble in distinct ways (10). In the β-carboxysome, the defined nucleation of Rubisco is mediated by binding with the structural proteins CcmM and CcmN (11–15). The shell is composed of protein hexamers, pentamers, and trimers, such as CcmK2, CcmK3, CcmK4, CcmL, CcmO, and CcmP in the model cyanobacterium *Synechococcus elongatus* 7942 (Syn7942) (16). The single-layer shell has been speculated to function as a semipermeable barrier to permit diffusion of bicarbonate into the carboxysome, prevent CO_2_ leakage, and likely exclude O_2_ (17). CcaA was proposed to localize in the inner shell surface and to dehydrate bicarbonate that diffuses through the shell to CO_2_ within the carboxysome lumen. These naturally occurring architectural features of carboxysomes permit generation of elevated levels of CO_2_ around Rubisco, thereby enhancing carbon fixation (18). In addition, the protein stoichiometry and organization of carboxysomes are regulatable, providing a means for physiological adaptation of the CO_2_-fixing organelles in response to environmental changes (19). At the subcellular context, proper positioning of carboxysomes has been suggested to be vital for equal separation of carboxysomes between daughter cells during cell division and maintenance of photosynthetic performance (20, 21).

Our current knowledge of the biosynthesis principles of carboxysomes remains primitive. Recent studies have suggested that the formation of β-carboxysomes proceeds via an inside-out manner (22, 23). Rubisco complexes first aggregate through binding with CcmM to form the enzyme core that appears as a liquid-like matrix (13, 14). The Rubisco-containing core structures are then encased by shell proteins, with the recruitment protein CcmN that interacts with CcmM, to form intact carboxysomes. This hierarchical assembly pathway suggests the significance of Rubisco condensate in carboxysome formation.

Rubisco in β-carboxysomes represents a plant-like, form 1B Rubisco and is a hexadecameric complex composed of eight large subunits (RbcL, 50 to 55 kDa) and eight small subunits (RbcS, 12 to 18 kDa), denoted as RbcL_8_S_8_ (3). RbcL subunits are arranged as a tetramer of antiparallel RbcL dimers to form RbcL_8_ as the main catalytic core, along with eight RbcS subunits that dock at the top and bottom to stabilize RbcL_8_ and tune Rubisco activity (24). The assembly of Rubisco represents a paradigm of chaperone-assisted processes. The folding of newly-synthesized RbcL requires the chaperonin proteins, GroEL and GroES (25), and the subsequent assembly of folded RbcL monomers into oligomers is triggered by binding with the chaperones Rubisco assembly factor 1 (Raf1) (26) and RbcX (27).

Although Raf1 is evolutionarily conserved, its importance in plants and cyanobacteria remains controversial. In maize, genetic deletion of *raf1* resulted in the depletion of Rubisco and plant lethality (28); overexpression of Rubisco subunits in combination with Raf1 could increase Rubisco protein content and activity (29). In addition, *Arabidopsis* Raf1 could promote the assembly of recombinant Rubisco in tobacco chloroplasts (30). The exact roles of Raf1 in cyanobacteria remain enigmatic (31). In vitro reconstitution experiments implied that Raf1 functions in stabilizing the antiparallel RbcL_2_ dimer (26). In-depth structural information of the RbcL-Raf1 complexes is important for elucidating the detailed mechanism in Raf1−Rubisco interactions and the actual function of cyanobacterial Raf1 in Rubisco assembly. Moreover, whether de novo carboxysome biogenesis requires external factors has remained elusive.

Here we investigated the structure of RbcL-Raf1 supercomplexes from Syn7942 and the roles of Raf1 in carboxysome biogenesis and cell physiology. Reconstruction and cryo-electron microscopy (cryo-EM) of the Syn7942 RbcL-Raf1 complex demonstrate that Raf1 is critical for assembly of Rubisco holoenzymes by mediating the formation of RbcL dimer and RbcL dimer–dimer interactions. Genetic deletion of *raf1* in Syn7942 leads to reductions in Rubisco protein content, cell growth, and CO_2_-fixing activity. Confocal microscopy and transmission electron microscopy (TEM) reveal that deletion of *raf1* impairs the formation of canonical carboxysomes and results in the generation of Rubisco-containing assemblies without shell proteins, a type of structural intermediate in carboxysome biogenesis, as well as a low abundance of small carboxysome-like structures with disordered interior packing. Our results provide a molecular view of the role of Raf1 in de novo β-carboxysome formation in Syn7942 and indicate the correlation between Raf1-mediated Rubisco assembly and carboxysome formation.

## Results

### Raf1 Is Important for Cyanobacterial Physiology.

**Fig. 1.**
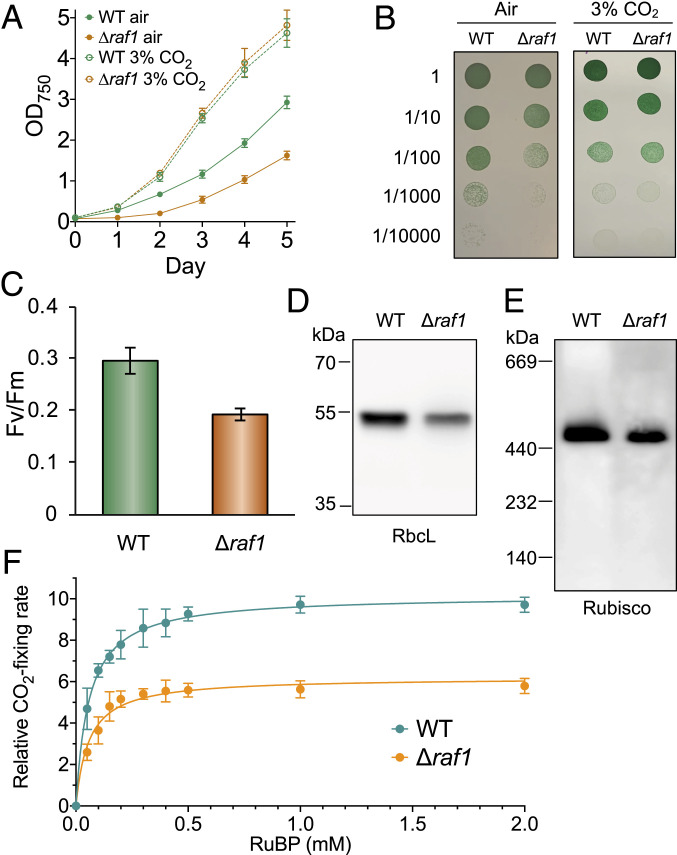
Cell physiology, Rubisco abundance, and CO_2_-fixing activity of the Syn7942 Δ*raf1* mutant. (*A*) Growth of the WT and Δ*raf1* mutant in ambient air and 3% CO_2_ monitored by OD_750_ measurement. Data are presented as mean ± SD for three independent cultures. (*B*) Dilution spot-plate growth of WT and Δ*raf1* strains in ambient air and 3% CO_2_. (*C*) Photosynthetic capacities (Fv/Fm) of WT and Δ*raf1*. Data are presented as mean ± SD for three biological replicates. (*D* and *E*) Comparison of the Rubisco amount in WT and Δ*raf1* by SDS-PAGE (*D*) and BN-PAGE (*E*), followed by immunoblot analysis using the anti-RbcL antibody. RbcL assemblies in Δ*raf1* exist predominantly in the form of L_8_S_8_, with the same molecular weight as those in WT. No RbcL_2_ or RbcL_8_ assemblies were detectable at the bottom of the Western blot image. (*F*) Kinetics of whole-cell carbon fixation activities of WT and Δ*raf1* strains, normalized by the total RbcL amounts. Data are presented as mean ± SD. Five independent cell cultures were analyzed. The maximum Rubisco activity and RuBP affinity *K*_m(RuBP)_ value were calculated by fitting to the Michaelis–Menten first-order rate equation. The maximum Rubisco activity of Δ*raf1* cells is ∼39% lower than that of WT cells; comparable mean *K*_m(RuBP)_ values were measured for WT (57 ± 11 µM; *n* = 5) and Δ*raf1* (58 ± 4 µM; *n* = 5).

To study the roles of Raf1 in cyanobacteria, a *raf1* deletion Syn7942 mutant (∆*raf1*) was generated (*SI Appendix*, Fig. S1*A* and *Materials and Methods*), and the genomic homogeneity and full deletion of *raf1* were confirmed by PCR analysis (*SI Appendix*, Fig. S1*B*). Growth assays illustrated that although surviving in air, the ∆*raf1* mutant exhibited a notable reduction in cell growth under ambient conditions; 3% CO_2_ could recover cell growth, indicating the defective CO_2_ metabolism of the Δ*raf1* strain (Fig. 1 *A* and *B*). In photosynthetic organisms, disruption of carbon fixation could ultimately result in damage to photosystem II (32). An ∼35% reduction in photosystem II activity (Fv/Fm) was measured in the Δ*raf1* mutant compared with the wild-type (WT) strain (Fig. 1*C*; *n* = 3). Sodium dodecyl sulfate polyacrylamide gel electrophoresis (SDS-PAGE) and immunoblot analysis using an anti-RbcL antibody revealed an ∼33% reduction in RbcL content in the soluble fraction of the total cell lysate in the ∆*raf1* mutant (sample loading was normalized against total protein amount; *n* = 3) compared with WT (Fig. 1*D*). Semiquantitative real-time PCR indicated that RbcL transcription was not affected by genetic deletion of *raf1* (*SI Appendix*, Fig. S1*C*), suggesting that the reduction in RbcL abundance led by *raf1* deletion is likely due to posttranslational regulation. Moreover, blue-native polyacrylamide gel electrophoresis (BN-PAGE) and immunoblot analysis of protein extracts from the ∆*raf1* cells showed only a single band corresponding to the Rubisco complex (L_8_S_8_) in WT (Fig. 1*E*), indicating that the Rubisco RbcL_8_S_8_ holoenzymes could still be formed in the absence of Raf1 but with low efficiency. The RbcL_8_S_8_ abundance exhibited an ∼30% drop (*n* = 3; Fig. 1*E*), consistent with the SDS-PAGE results (Fig. 1*D*).

Whole-cell radiometric carbon fixation assays normalized by the total amount of RbcL revealed that Rubisco in the ∆*raf1* mutant has a ∼39% reduction in the maximum CO_2_-fixing capacity (*n* = 5) and comparable affinities to the substrate ribulose 1,5-bisphosphate (RuBP) (*K*_m(RuBP)_ = 58 ± 4 µM for ∆*raf1* vs. *K*_m(RuBP)_ = 57 ± 11 µM for WT; *n* = 5) (Fig. 1*F*). Collectively, our results demonstrate that the absence of Raf1 led to decreases in cell growth, Rubisco protein content, and CO_2_-fixing capacity, and that Raf1 is important but not compulsory for the formation of Rubisco RbcL_8_S_8_ holoenzymes in Syn7942 and cell survival in air.

### Structural Analysis of the RbcL-Raf1 Complex.

**Fig. 2.**
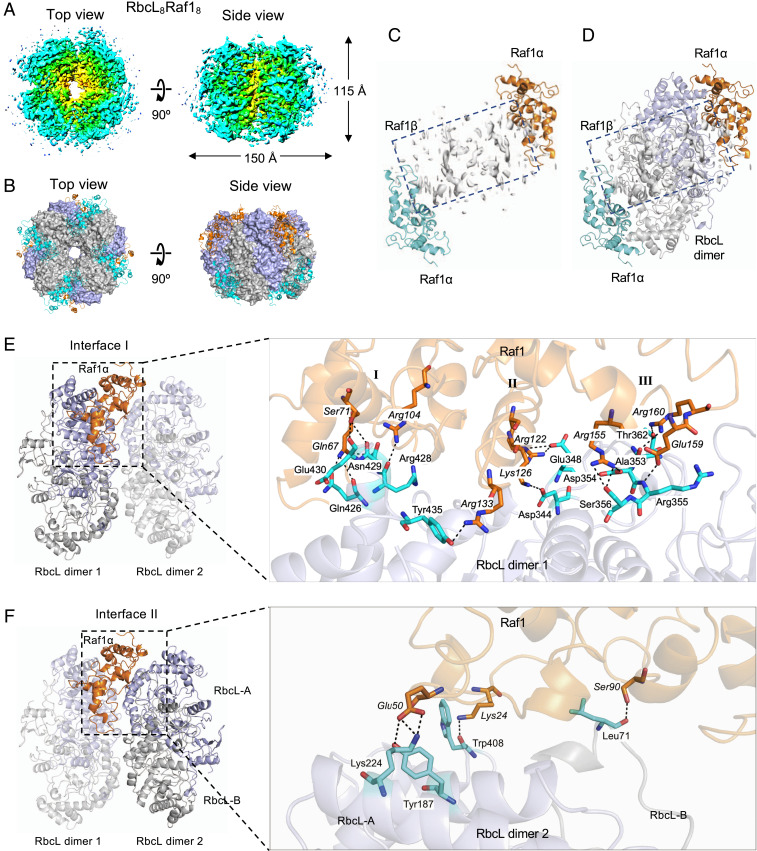
Structural analysis of the Syn7942 RbcL-Raf1 complex by cryo-EM. (*A*) 3D cryo-EM density map of the RbcL_8_-Raf1α_8_ complex (EMD ID code 10235) from the top view (*Left*) and the side view (*Right*). (*B*) Structure of the RbcL_8_-Raf1α_8_ complex (PDB ID code 6SMH) from the top view (*Left*) and the side view (*Right*). Raf1 domains are shown in cartoon, and RbcL subunits were shown in surface representation. (*C* and *D*) Structure of the Syn7942 Raf1α domains in the dimer on its own (*C*) and associated with an antiparallel RbcL dimer (*D*) derived from the cryo-EM structure of RbcL_8_-Raf1α_8_. The two N-terminal Raf1α domains are presented in orange and blue. The electron density indicates the orientation of the flexible C-terminal Raf1β domain (boxed). The Raf1β positions at the equatorial front face of each RbcL dimer and the two α-domains bracket the RbcL dimer. The Raf1β structure could not be well resolved due to the limited cryo-EM resolution. (*E* and *F*) Each Raf1α domain shows two interacting interfaces with the neighboring RbcL dimers, with the interface I between the α-domain and the RbcL dimer 1 (*E*), which includes three contact regions (I, II, and III), and the interface II between the α-domain and the neighboring RbcL dimer 2 (*F*). Close-up views of the interfaces reveal the interacting amino acid residues of Raf1α (orange) and RbcL (light blue), as well as salt bridges and van der Waals contacts indicated by dashed lines. Interacting residues are labeled in italics for Raf1α and in roman type for RbcL.

Previous low-resolution structural analysis of the Raf1-RbcL complex has indicated that Raf1 assists in the formation of RbcL dimers that are competent to form holoenzyme (26). To elucidate how Raf1 mediates Rubisco assembly, we obtained a higher-resolution structure of the Syn7942 RbcL-Raf1 supercomplex expressed in *Escherichia coli* using cryo-EM (*SI Appendix*, Fig. S2). The cryo-EM density map of the RbcL-Raf1 supercomplex was solved to 4.28-Å resolution (16,022 particles; *SI Appendix*, Table S1 and Fig. S2), with variable local resolutions from the central to the peripheral regions of the complex (Fig. 2 *E* and *F*). The model of the Syn7942 RbcL-Raf1 structure was reconstituted and refined using the crystal structures of Syn7942 Rubisco (Protein Data Bank [PDB] ID code 6HBC) and *Anabaena* sp. PCC 7120 RbcL-Raf1 complex (PDB ID code 6KKM).

The Syn7942 RbcL-Raf1 complex possesses a four-fold symmetry, with a diameter of 150 Å and a height of 115 Å (Fig. 2*A*). The core is composed of eight RbcL subunits that arrange as a tetramer of antiparallel dimers (RbcL_8_) and four Raf1 dimers located at the peripheral surface of the RbcL_8_ core, sitting at the interface cleft between two neighboring RbcL dimers (Fig. 2*B* and *SI Appendix*, Fig. S3). Raf1 contains an N-terminal α-helical domain (Raf1α) and a C-terminal β-sheet dimerization domain (Raf1β), with a flexible linker between Raf1α and Raf1β (26). Raf1α competes with RbcS in binding with RbcL, as both of them interact with the similar regions of the RbcL_8_ core (Fig. 2*B*). Two Raf1α in each Raf1 dimer are interlocked by the Raf1β dimer (Fig. 2*C*; electron density is boxed), clipping the RbcL antiparallel dimer at the top and bottom sides (Fig. 2*D* and *SI Appendix*, Fig. S3). The Raf1β locates at the equator of the RbcL dimer (Fig. 2*D*). The atomic structure of the Syn7942 Raf1β segment could not be resolved at our resolution, likely due to the structural flexibility of this domain.

Structural analysis of the RbcL_8_-Raf1_8_ complex revealed that Raf1α interacts with three RbcL subunits of two adjacent RbcL dimers via mainly salt bridges and hydrogen bonds. Two interaction interfaces were identified between Raf1α and its adjacent RbcL dimers. Raf1α was found to bind to the RbcL dimer 1 on interface I through multiple contacts located in three distinct regions possessing a few α-helixes (Fig. 2 *E* and *F*). These contact residues of Raf1α include Gln67, Ser71, and Arg104 (region I); Arg122, Lys126, and Arg133 (region II); and Arg155, Glu159, and Arg160 (region III). Previous mutation analyses have consistently suggested the binding of Raf1 Arg104 and Lys126 residues to RbcL (26). On interface II, where Raf1α interacts with the adjacent RbcL dimer 2, Lys24 and Glu50 interact with Tyr187 and Lys224 residues of RbcL-A, while Ser90 binds to the Leu71 residue of RbcL-B (Fig. 2*F*). This structural analysis revealed explicitly that Syn7942 Raf1 is important not only for the formation and stabilization of RbcL dimers, but also for the mediation of RbcL dimer–dimer interaction to facilitate the assembly of Rubisco holoenzymes. Sequence alignment showed that these binding residues are conserved among Raf1 homologs from cyanobacteria, green algae, and plants (*SI Appendix*, Fig. S4), indicative of the general binding principles of the RbcL-Raf1 complex.

### Carboxysome Formation Is Impaired in the Absence of Raf1.

**Fig. 3.**
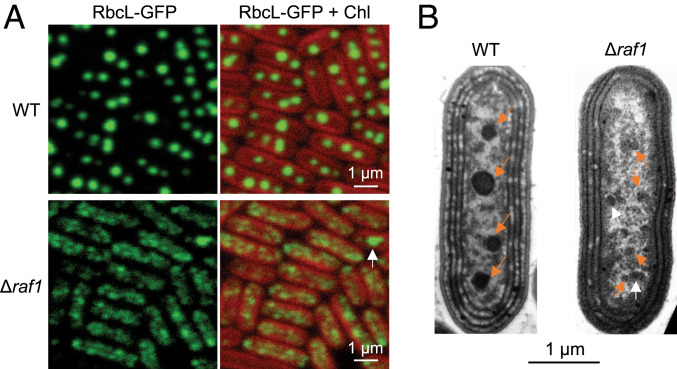
Carboxysome formation is impaired in the Syn7942 Δ*raf1* mutant. (*A*) Confocal microscopy images of RbcL-GFP in WT (*Top*) and Δ*raf1* mutant (*Bottom*). Green signals represent RbcL labeled with GFP. It shows the carboxysome structures in the WT cells, which is completely disturbed in the Δ*raf1* mutant cells, where GFP signals aggregate into small assemblies in the cytosol. Red signals represent the chlorophyll (Chl) autofluorescence from thylakoid membranes. The white arrow indicates a larger fluorescent focus that likely represents a low abundance of small carboxysome-like structures formed in Δ*raf1* cells. (*B*) Thin-section TEM images of Syn7942 WT (*Left*) and Δ*raf1* mutant (*Right*). Orange arrows indicate the carboxysomes in the WT and assembly intermediates in the Δ*raf1* cells. Occasionally, larger carboxysome-like structures can be observed (white arrow).

Given that Rubisco assembly and nucleation are prerequisites for carboxysome biogenesis (22, 23), we wondered whether carboxysome formation is affected without Raf1. To address this question, a Δ*raf1*/RbcL-GFP mutant was generated to monitor the formation process of carboxysomes in vivo (*SI Appendix*, Fig. S5*A*). In the RbcL-GFP strain, three or four fluorescence foci were spaced along the long axis of the cell (Fig. 3*A*), typical for carboxysome biogenesis and positioning in Syn7942 (20–22, 33). In contrast, no canonical distribution of carboxysomes was discerned in Δ*raf1*; instead, numerous smaller fluorescent foci with lower signal intensities were observed with a random distribution in the cytosol (Fig. 3*A*). Quantification of the total fluorescence intensity per cell based on confocal images (acquired under the same microscopic settings) revealed that Rubisco abundance per cell was reduced by ∼40% by deletion of *raf1* (*n* = 250; *SI Appendix*, Fig. S5*B*), in agreement with immunoblot results (Fig. 1 *C* and *D*). Thin-section TEM revealed small electron-dense structures in the Δ*raf1* cells that were structurally distinct from the polyhedral carboxysomes observed in WT (Fig. 3*B*, arrows). These structures were also observed by thin-section TEM in the Δ*raf1*/RbcL-GFP cells (*SI Appendix*, Fig. S5*C*).

Complementation experiments showed the recovery of carboxysome formation when expressing plasmid-encoded Syn7942 Raf1 in the Δ*raf1/RbcL-GFP* mutant (*SI Appendix*, Fig. S6). The level of this recovery appears to be dependent on the Raf1 content. Collectively, our results demonstrate the defective formation of intact carboxysomes caused by loss of Raf1.

The carboxysome core organizing protein CcmM exists in two forms: full-length CcmM (M58) and truncated CcmM (M35). M58 contains the N-terminal CA-like domain that interacts with CcmN to recruit the shell proteins for encapsulation and the C-terminal repeat Rubisco small subunit-like (SSUL) domains that are involved in Rubisco complex packing. M35 lacks the CA-like domain and contains only the SSUL domains (11, 12). The stoichiometric ratio of M58 and M35 has been proposed to be key for carboxysome formation (12); therefore, we tested the relative ratio of M58/M35 in the soluble fraction of the WT and *Δraf1*. Immunoblot analysis against an anti-CcmM antibody revealed a greater than threefold increase in the M58/M35 ratio in *Δraf1* compared with WT (*SI Appendix*, Fig. S7). Coincidentally, small electron-dense structures have been reported previously in the Syn7942 mutant with an elevated M58/M35 ratio (12), similar to the small protein assemblies identified in the Δ*raf1* mutant (Fig. 3*B* and *SI Appendix*, Fig. S5*B*). Overall, the confocal, TEM, and immunoblot results imply that the absence of Raf1 resulted in impaired carboxysome formation and generation of small assembly intermediates in Δ*raf1,* probably by altering the M58/M35 ratio.

### The Rubisco-Containing Assembly Intermediates Exclude Shell Encapsulation.

**Fig. 4.**
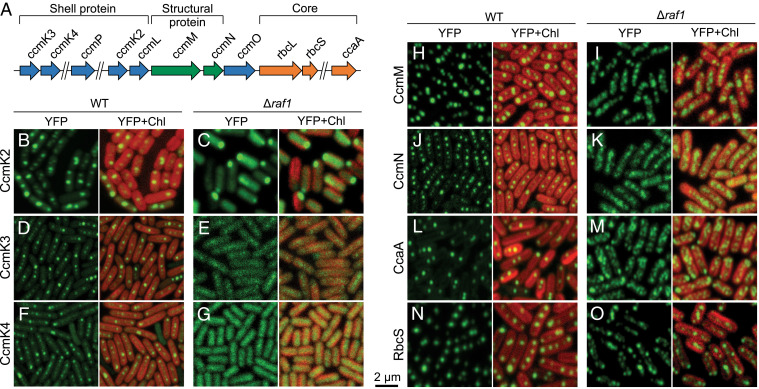
Composition of the carboxysome assembly intermediates induced by *raf1* deletion in Syn7942. (*A*) Genomic locations of genes encoding carboxysome components in Syn7942. Shell protein genes are labeled in blue, internal catalytic components, in orange; and internal linker components, in green. (*B*–*O*) Confocal microscopy images of carboxysome components labeled with YFP in the WT and the Δ*raf1* mutant. Green signals represent YFP-tagged carboxysome proteins, and red signals represent chlorophyll (Chl) autofluorescence. Signals of the shell proteins CcmK2, K3, and K4 exhibit the typical carboxysome distribution in WT cells (*B*, *D*, and *F*) but appear to be evenly distributed in the cytosol of Δ*raf1* cells (*C*, *E*, and *G*). Note that the fluorescent puncta of CcmK2-YFP located at the cell pole of Δ*raf1* is likely due to overaccumulation of unassembled CcmK2 proteins (*C*). In contrast, in the Δ*raf1* mutant, the distribution of the internal linker proteins CcmM and CcmN as well as that of the cargo proteins CcaA and RbcS resembles RbcL that aggregate into small assemblies in the cytosol (*I*, *K*, *M*, and *O*), distinct from their carboxysome-like distribution patterns observed in WT (*H*, *J*, *L*, and *N*) and the cytosolic distribution of CcmK2, K3, and K4 in Δ*raf1* (*C*, *E*, and *G*).

Previous work has shown that in the Syn7942 *ccmM*-null mutant that cannot generate carboxysomes, RbcL proteins are evenly distributed throughout the cytosol (22), distinct from the patchy RbcL-GFP distribution observed in the Δ*raf1* mutant. This suggests that in the absence of Raf1, Rubisco could aggregate with each other or interact with other carboxysome proteins (such as the organizing proteins and interior enzymes, or shell proteins) to form protein assemblies. To test this hypothesis, we generated a series of Syn7942 Δ*raf1* mutants in which enhanced yellow fluorescence protein (YFP) were systematically tagged to various carboxysome proteins, including the shell proteins CcmK2, CcmK3, and CcmK4; the structural proteins CcmM and CcmN that are essential for packing of Rubisco complexes (12, 14, 15); and the interiors CcaA and RbcS (Fig. 4*A*). Fluorescence tagging was placed at the native chromosomal locus under the control of their native promoters to permit expression of the fluorescently tagged proteins in context and at physiological levels.

Live-cell fluorescence imaging identified the carboxysome formation and typical localization in all YFP-tagged strains in the WT background (Fig. 4 *B*, *D*, *F*, *H*, *J*, *L*, and *N*), consistent with our previous observations (19). In the ∆*raf1* mutant, in contrast, CcmK2, CcmK3, and CcmK4 diffused predominantly throughout the cytosol (Fig. 4 *C*, *E*, and *G*), whereas CcmM, CcmN, CcaA, and RbcS exhibited patchy distribution similar to that of RbcL-GFP observed in Δ*raf1* (Fig. 3*A*). Furthermore, confocal images of the Δ*raf1*/CcmM-YFP/RbcL-CFP and Δ*raf1*/CcmK4-YFP/RbcL-CFP double-labeled mutants confirmed the colocalization of RbcL and CcmM, as well as the distinct subcellular locations of RbcL and CcmK4 induced by *raf1* deletion (*SI Appendix*, Fig. S8).

Along with its cytosolic distribution, CcmK2-YFP has a propensity to form large puncta at the cell poles (Fig. 4*C*), probably due to overaccumulation of unassembled CcmK2 proteins, as shown in previous studies (22). We further examined the relative abundance of CcmK2 in the supernatants and pellets after centrifugation at 40,000× *g* (following a centrifugation at 10,000× *g*; *Materials and Methods*). Most of the CcmK2 proteins in WT cells were in the 40,000× *g* pellet, which contains enriched intact carboxysomes (*SI Appendix*, Fig. S9). In contrast, in the Δ*raf1* mutant, CcmK2 proteins were mainly in the supernatant, validating that most shell proteins were not recruited by the Rubisco core assemblies for encapsulation. Taken together, our results demonstrate that CcaA, RbcS, CcmM, and CcmN colocalize with Rubisco within the formed macromolecular assemblies in Δ*raf1*, whereas shell encapsulation of the core assembly intermediates is impaired by the absence of Raf1.

### Isolation and Structural Characterization of the Assembly Intermediates.

**Fig. 5.**
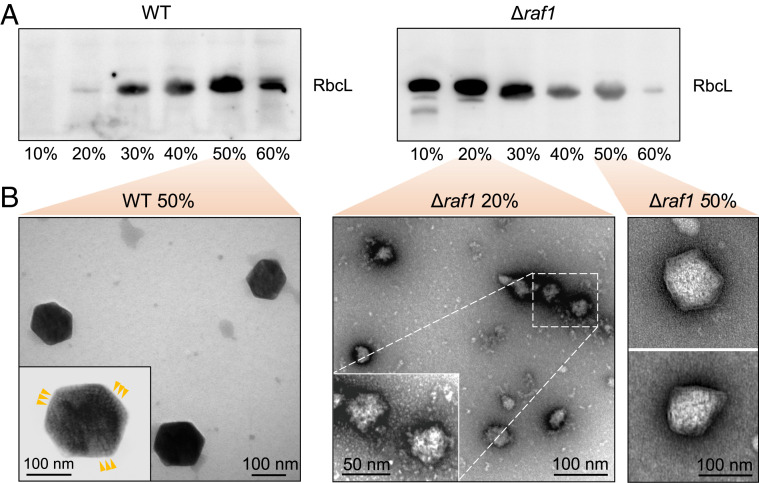
Characterization of the carboxysome assembly assemblies. (*A*) Relative Rubisco amount in each sucrose gradient fraction achieved during carboxysome isolation from the WT (*Left*) and Δ*raf1* mutant (*Right*), as revealed by SDS-PAGE and immunoblot analysis using an anti-RbcL antibody. (*B*) Negative-staining TEM images of intact carboxysomes in the 50% fraction from WT (*Left*) and assembly intermediates in the 20% and carboxysome-like structures in the 50% fractions from the Δ*raf1* cells (*Right*). (*Insets*) Zoom-in views representing the structures and protein organization of an intact carboxysome from the WT and the assembly intermediates from the Δ*raf1* mutant. Arrows indicate the ordered Rubisco arrays observed in the WT carboxysomes.

To further characterize the Rubisco-containing assemblies, we isolated carboxysomes and assembly intermediates from WT and Δ*raf1* cells, respectively, following the method modified from the previous work (9). Rubisco proteins from WT were mainly enriched in the 40 to 60% sucrose fractions (Fig. 5*A*), implicating the formation of intact carboxysomes. In contrast, Rubisco proteins from *Δraf1* were determined mainly between the 10% and 30% sucrose fractions (Fig. 5*A*), indicating a lower molecular mass of the resulting supramolecular assemblies compared with that of intact carboxysomes. Rubisco was also identified in the 40 to 60% sucrose fractions of Δ*raf1*, although at a lower abundance compared with WT (Fig. 5*A*).

Samples from the 20% and 50% sucrose fractions of Δ*raf1* were both characterized by negative-staining TEM. In the 20% fraction of Δ*raf1*, Rubisco-containing assemblies were seen to have no clear physical boundary and to have disordered protein organization, manifestly differing from the typical polyhedral shape of WT carboxysomes with regular Rubisco packing and shell encapsulation (Fig. 5*B*) (9). In the 50% sucrose fraction of Δ*raf1*, we observed a small number of carboxysome-like structures encapsulated by outer shells (Fig. 5*B*). Distinct from the WT carboxysomes (151.6 ± 23.1 nm in diameter), these carboxysome-like particles exhibit aberrant structures, with the reduced size (105.9 ± 15.18 nm) and irregular polyhedral shell shapes (Fig. 5*B* and *SI Appendix*, Fig. S10). Another intriguing feature is the disordered packing of Rubisco enzymes, consistent with the structures of Rubisco-containing core assemblies at the 20% fraction. Similar carboxysome-like structures were also occasionally visualized in thin-section TEM of the Δ*raf1* cells and negative-staining TEM of the 50% fraction of the Δ*raf1*/RbcL-GFP mutant (*SI Appendix*, Fig. S11). The presence of the carboxysome-like structures was also indicated in confocal images (Fig. 3*A*, white arrow). Consistent with this, a small fraction of CcmK2 proteins were detected in the pellet of the Δ*raf1* cell extracts (*SI Appendix*, Fig. S9), indicating the formation of carboxysome structures with shell encapsulation, although some CcmK2 proteins may self-aggregate. These carboxysome-like structures, although structurally distinguishable from canonical WT β-carboxysomes and having a low content in cells, may provide carbon fixation activities to ensure the survival of the Δ*raf1* cells in air (Fig. 1).

### In Vivo Diffusion Dynamics of the Assembly Intermediates.

Mature carboxysomes possess defined localization and constrained diffusion dynamics in cyanobacteria due to interactions with specific cellular components (19–21, 33, 34). The resulting carboxysome assembly intermediates in Δ*raf1* exhibit different subcellular positioning compared with the canonical carboxysome distribution (Fig. 3). To determine whether the assembly intermediates are also spatially constrained, time-lapse live-cell fluorescence imaging was conducted to track the diffusion dynamics of the carboxysome intermediates in the Δ*raf1* cells (*SI Appendix*, Fig. S12 and Movie S1). In the RbcL-GFP cells, carboxysomes underwent a constrained oscillation within the local regions, and crossover between carboxysomes was rarely seen, similar to previous observations (19–21). In contrast, the fluorescence foci in the Δ*raf1*/RbcL-GFP cells showed an ∼1.3-fold increase in the mean diffusion velocity (29.3 nm∙s^−1^ for Δ*raf1*/RbcL-GFP and 12.6 nm∙s^−1^ for RbcL-GFP) and a greater motional area compared with the carboxysomes in the RbcL-GFP cells (*SI Appendix*, Fig. S12). The distinct diffusion behaviors signified the interrupted interaction between the generated assembly intermediates and the partners that determine the subcellular positioning of carboxysomes (20, 35), indicating the requirement of shell proteins for carboxysome segregation.

### Raf1 May Function in an Antagonistic Way with RbcX in Cyanobacterial Rubisco Assembly.

Raf1 is conserved in all photosynthetic organisms that contain RbcX, another Rubisco assembly chaperone, and has been found to have coevolved with RbcL in plants, algae, and cyanobacteria (30). Although differing in protein structure, both Raf1 and RbcX play roles in Rubisco assembly by promoting RbcL dimerization (3). In Syn7942, RbcX is not essential for cell growth (11, 36), leading to the assumption that Raf1 may function redundantly with RbcX in vivo. To examine the functional correlation between Raf1 and RbcX in vivo, we generated the Δ*raf1*/Δ*rbcX* and Δ*raf1*/Δ*rbcX*/RbcL-GFP Syn7942 mutants (*SI Appendix*, Fig. S13*A*). These mutants were able to survive in air. Confocal images showed that the defective carboxysome formation led by *raf1* deletion could be partially recovered in the Δ*raf1*/Δ*rbcX*/RbcL-GFP mutant (*SI Appendix*, Fig. S13*B*). Likewise, thin-section TEM of the Δ*raf1*/Δ*rbcX* cells showed the reoccurrence of carboxysome-like structures with clearly defined boundaries in the cytosol (*SI Appendix*, Fig. S13*C*). These results do not support the functional redundancy of Raf1 and RbcX in vivo. In addition, depletion of *rbcX* has been shown to lead to an increase in Rubisco content in Syn7942; carboxysomes were still formed in Δ*rbcX*, but exhibited an increased size and reduced copy numbers per cell (36). These observations are sharply opposite to the effects of *raf1* deletion. Interestingly, in vitro studies have indicated that RbcX plays a role in Rubisco evolvability by preventing mutations from forming holoenzymes compatible with functionality (37).

Taken together, the experimental results suggest that Raf1 and RbcX may functionally act in an antagonistic way in mediating Rubisco assembly in Syn7942. Raf1 appears to take the predominant role in this regulatory process, as deletion of *raf1* could cause the defective formation of carboxysomes. The detailed mechanisms underlying the assistance of Raf1 and RbcX to Rubisco assembly and β-carboxysome biogenesis merit further investigation.

## Discussion

In this work, we conducted structural and functional analyses of the Rubisco assembly chaperone protein Raf1 in vitro and in vivo and found that Raf1 is important for de novo Rubisco assembly and carboxysome formation in Syn7942. Our study provides evidence supporting the role of Raf1 in β-carboxysome biogenesis. The molecular basis of assembly factor-mediated carboxysome formation may be extended to other bacterial microcompartments and inform bioengineering of functional carboxysomes in heterologous hosts.

Our cryo-EM results for the RbcL-Raf1 supercomplex provide insight into the chaperonin-assisted Rubisco assembly process. In cyanobacteria, the Rubisco core complex RbcL_8_, the tetramer of antiparallel RbcL dimers, is formed with the assistance of chaperones Raf1 and RbcX, and acts as a harbor to dock key interacting partners at different binding sites, as revealed by the superimposed structures of RbcX (PDB ID code 2WVW), CcmM (PDB ID code 6HBC), RbcS (PDB ID code 6HBC), and RbcL_8_-Raf1_8_ (PDB ID code 6SMH; this study) (*SI Appendix*, Fig. S14). The SSUL modules of CcmM bind in a cleft between RbcL dimers at the equator of Rubisco to link Rubisco complexes into a network and induce the liquid-like Rubisco matrix (14). RbcX_2_ contacts two RbcL monomers within the RbcL dimer; the central cleft of RbcX_2_ is close to the C terminus of RbcL, and each RbcX_2_ also interacts with the N terminus of the adjacent RbcL monomer, thereby stabilizing RbcL dimers (27). RbcS plays important roles in stabilizing the RbcL_8_ structure and improving Rubisco activity (38). Raf1 competes with RbcS for binding to RbcL at the top and bottom of the Rubisco cylinder. Our cryo-EM analysis of RbcL_8_-Raf1_8_ elucidates that Syn7942 Raf1 functions in stabilizing not only individual RbcL dimers, but also dimer–dimer interactions, thereby facilitating formation of the RbcL_8_ core structure (Fig. 2). The multivalent nature of Rubisco and its capacity to bind with multiple molecules may reflect the intricate tuning mechanism adopted by the Rubisco enzyme to define and modulate its conformation and function.

**Fig. 6.**
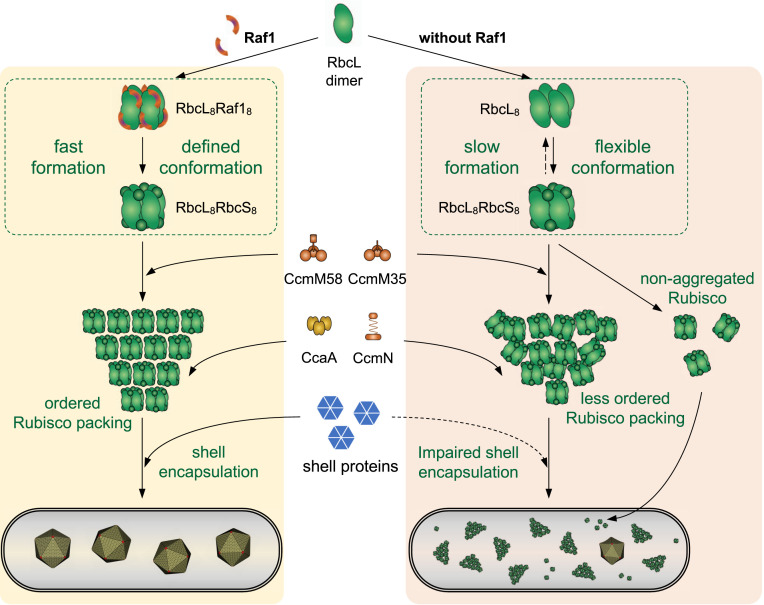
Schematic model of Raf1-mediated carboxysome formation in Syn7942. Raf1 facilitates the assembly of Rubisco and Rubisco assemblies. The absence of Raf1 results in slow and inefficient assembly and labile conformations of Rubisco L_8_S_8_ holoenzymes, leading to less well-defined Rubisco L_8_S_8_ structures and an imbalance of carboxysome protein stoichiometries, which may alter the binding of CcmM to Rubisco and protein packing in the Rubisco:M35 condensate. These changes could result in the formation of small and disordered Rubisco core assemblies and interfere with shell encapsulation. As a consequence, the Δ*raf1* cells produce a large number of assembly intermediates without shells along with a low abundance of small carboxysome-like structures and free Rubisco.

Following the “inside-out” pathway (22, 23), β-carboxysome biogenesis is initiated by stringent Rubisco packing and nucleation, which trigger shell recruitment and construction of functional and intact carboxysomes. Our data lead to the following speculative model of the role of Raf1 in β-carboxysome biogenesis (Fig. 6). The assembled RbcL_8_ could permit the capping of RbcS to form RbcL_8_S_8_ and the interactions with other key players, such as CcmM (14) (Fig. 4). In the absence of Raf1, a small amount of RbcL_8_ could still form, probably at a slower rate and with a labile structure, leading to the generation of low-abundance RbcL_8_S_8_ in the Δ*raf1* mutant (Fig. 1). The inefficient formation and labile conformation of Rubisco holoenzymes may result in an imbalance of carboxysome protein stoichiometries (i.e., M58:M35; *SI Appendix*, Fig. S7) and an undefined docking number of the CcmM35 SSUL domains to specific sites of Rubisco. As a consequence, small and disordered M35:Rubisco assemblies are prone to form (Fig. 5*B*), which permit the recruitment of CcmM58, CcmN, and CcaA but impair proper shell encapsulation, which is possibly size-dependent. In line with this, there are concomitant increases in the contents of shell proteins in the cytoplasm of the *Δraf1* cells (Fig. 3 and *SI Appendix*, Fig. S9). The increase in the M58:M35 ratio (*SI Appendix*, Fig. S7) is also consistent with the decrease in the interior volume (mediated predominantly by CcmM35) and the relative increase in the core surface area (accommodating CcmM58). A combination of these effects eventually results in the formation of a large number of assembly intermediates without shells and a small number of carboxysome-like structures, both exhibiting the characteristic reduced size and disordered interior organization. The Raf1-mediated Rubisco assembly and carboxysome formation appear to be dependent on the abundance of Raf1 in cells (*SI Appendix*, Fig. S6). Our model provides further support for the “inside-out” model of β-carboxysome assembly, and indicates that the volume, protein concentration, and organization of the Rubisco core assemblies are crucial for proper shell encapsulation and carboxysome formation.

Characterization of the assembly intermediates generated in the *Δraf1* mutant provides a means for understanding carboxysome biogenesis in molecular detail. Mature carboxysomes possess highly ordered Rubisco lattice inside the compartment and have regular distribution patterns in cyanobacteria cells (9, 20–22, 33). In the ∆*ccmM* mutant, Rubisco proteins appear to diffuse throughout the cytosol and do not form carboxysome structures, whereas in the ∆*ccmK2* mutant, Rubisco proteins are prone to aggregate and form “procarboxysomes” at the cell poles, which are composed of Rubisco and CcmM (22). The assembly intermediates generated in the absence of Raf1 differ from any of the foregoing structures, given their distinguishable size, composition, structural heterogeneity, and subcellular positioning. These protein aggregates, composing primarily Rubisco, CcmM, CcaA, and CcmN, have smaller dimensions, resembling the electron-dense structures observed in the Syn7942 mutant cells with the increased stoichiometry of M58/M35 (12). Similar Rubisco assemblies have been identified in a marine cyanobacterium (39). The disordered protein organization and reduced cargo size might not be sufficient to trigger shell encapsulation and the concomitant formation of intact carboxysomes (23).

The significance of carboxysomes in improving carbon fixation has been proposed to be related to their naturally occurring properties, including self-assembly, shell permeability, condensation of Rubisco enzymes, and colocalization of CA. The survival of the Δ*raf1* mutant in air without formation of canonical carboxysomes could be explained by two possibilities. One possibility is that the existence of shell-encapsulated carboxysome-like structures, even in low abundance and with defective structures, would inevitably contribute to certain levels of carbon fixation activity and support the Δ*raf1* strain to survive in air. Another possibility is that the assembly aggregates composed of Rubisco, CcaA, CcmM, and CcmN may provide a metabolically favorable microenvironment (i.e., specific protein–protein interactions, oxidized conditions) for the catalytic activities of Rubisco and CA, resulting in certain levels of CO_2_-fixing activities. This may be particularly essential for those cells that do not produce small carboxysome-like structures. Of note, a previous study reported that a carboxysome-less pseudorevertant Δ*ccmM* Syn7942 mutant could survive in air (40). Further investigations are needed to tackle this question.

Introducing the cyanobacterial CCM, including functional bicarbonate transporters and carboxysomes, into plant chloroplasts is a recognized strategy to boost photosynthesis and crop productivity (6, 7, 41). Recently, a simplified α-carboxysome from cyanobacteria was successfully assembled in tobacco chloroplasts (42). In contrast, little progress has been made in constructing functional β-carboxysomes, which naturally encapsulate plant-like Rubisco, in plants. The coevolution of Raf1 with RbcL in cyanobacteria and plants (30) implies that plant Raf1 might not be compatible with cyanobacterial Rubisco assembly and carboxysome construction in chloroplasts. Expressing the compatible Raf1 and Rubisco could be an important consideration for the engineering of intact, functionally active carboxysomes in heterologous organisms.

## Materials and Methods

### Bacterial Strains, Growth Conditions, and Physiology.

The cyanobacterium Syn7942 was maintained on solid BG11 medium (43) at 30 °C with constant illumination of a light intensity of 30 to 40 µmol photons m^−2^ s^−1^ provided by LED tubes. Liquid cultures were grown at 30 °C with constant illumination in BG11 medium in culture flasks with constant shaking. Cultures were grown in air without an additional CO_2_ source, except for the growth assays under CO_2_ treatment, in which Syn7942 cultures in the growth incubators were aerated with 3% (vol/vol) CO_2_. The cell growth curve was performed with a starting OD_750_ of 0.1. Chlorophyll fluorescence kinetics were measured on an AquaPen-C fluorometer (Photon Systems Instruments) using cells diluted to a Chl-a concentration of 1 to 2 µg mL^−1^ and dark-adapted for 3 min. Where appropriate, apramycin (Apra), kanamycin (Kan), or spectinomycin (Spec) (44) was added to the medium at a final concentration of 50 µg mL^−1^, 50 µg mL^−1^, or 25 µg mL^−1^, respectively.

### Generation of Constructs and Syn7942 Transformation.

The oligonucleotides used in cloning and segregation analysis are listed in *SI Appendix*, Table S2. To make the *raf1* deletion construct, a gene fragment containing the *raf1* (*synpcc7942_0833*) ORF and its upstream and downstream sequences was first amplified from the Syn7942 genomic DNA using the primers raf1F and raf1R and then cloned into the pGEM-T Easy vector (Promega), yielding pGEM-raf1. Then the gene fragment containing the Spec resistance gene and the flanking linker regions was amplified from the plasmid pIJ778-Spec (36) using the primers raf1koF and raf1koR and was used to replace *raf1* in pGEM-raf1 to generate a pGEM-raf1ko construct using homologous recombination (45, 46). A fragment containing a Kan resistance gene and flanking linker regions was amplified from pIJ786-Kan using primers raf1koF and raf1koR, and the fragment was used to replace the *raf1* gene in pGEM-raf1. All constructs were transformed into WT Syn7942 and RbcL-GFP mutant (33, 36) to generate raf1 KO mutants as described previously (47). The segregation of insertions was tested with primers raf1kosegF and raf1kosegR, and the expected size was shown in *SI Appendix*, Fig. S1. For the complementation experiment, the Syn7942 *raf1* was amplified using primers pAMraf1F and pAMraf1R from Syn7942 genomic DNA and inserted into a modified pAM2991 vector (48) with Kan resistance (49) at the EcoRI and BamHI sites under the control of isopropyl β-d-1-thiogalactopyranoside (IPTG)-inducible Ptrc promoter. The vector thus generated was then transformed into Δ*raf1*/RbcL-GFP cyanobacteria mutant cells and expressed in the presence of 100 μM IPTG.

The generation of fluorescently tagged carboxysome proteins has been reported in our recent studies (19, 33). All fluorescence-fusion mutants were generated following the REDIRECT protocol (45), by inserting the *eyfp*:*apramycin* DNA fragment into the 3′-end of individual carboxysome genes based on homologous recombination. Therefore, fluorescence tagging was placed at the native chromosomal locus under the control of their native promoters, permitting expression of the fluorescently tagged proteins in context and at physiological levels (50, 51).

For the constructs used in our in vitro cryo-EM analysis, the genes encoding GroES/EL (*synpcc7942_2313/2314*), RbcL (*synpcc7942_1426*), and Raf1 (*synpcc7942_0833*) were amplified from the genomic DNA of Syn7942. The GroES/EL genes were cloned into the pCDFDuet vector using primers GroES/EL_F and GroES/EL_R to generate the plasmid denoted pCDFDuet-GroEL-GroES. RbcL and Raf1 were cloned into the pET19b vector using primers p19RbcLF, p19RbcLR, p19Raf1F, and p19Raf1R to generate the construct denoted as pET19b-RbcL-Raf1.

### Protein Expression and Purification from *E. coli.*

The plasmids pCDFDuet-GroEL-GroES and pET19b-RbcL-Raf1 were cotransformed into the *E. coli* strain BL21 DE3 (Invitrogen). The transformant grown to an OD_600_ of 0.6 to 0.8 at 37 °C in Luria-Bertani (LB) medium was induced with 0.2 mM IPTG, and grown for additional ∼20 h at 16 °C. The cells were harvested and resuspended in the lysis buffer containing 50 mM Tris⋅HCl (pH 8.0), 20 mM NaCl, 5 mM MgCl_2_, and disrupted by ultrasonication. The supernatant obtained after centrifugation (17,300× g, 30 min) was applied to a Ni-IMAC column (GE Healthcare) to capture the His-fusion complex. The target protein was eluted with the lysis buffer supplemented with 300 mM imidazole. Afterward, the protein complex was further purified by size-exclusion chromatography using Superdex 200 (GE Healthcare). Fractions containing the complex were combined and concentrated using Amicon Ultra 100 kDa. All purification procedures were performed at 4 °C. The purity of proteins was assessed by gel electrophoresis. The molecular weight and the stoichiometry of the protein complex were determined by multiangle static light scattering.

### RNA Isolation, cDNA Synthesis, and Semiquantitative RT-PCR.

Syn7942 cells were collected by centrifugation (6,000× g, 5 min) in 50-mL centrifuge tubes and concentrated in 1 mL growth medium and transferred to a 1.5-mL microcentrifuge tube. The cells were recentrifuged (10,000× g, 1 min), and the pellet was used for total RNA isolation using TRIzol reagent protocol (Invitrogen). The RNA was digested with 4 units of DNase (RQ1 RNase-free DNase, Promega) according to the manufacturer’s instructions before cDNA synthesis to avoid amplifying genomic sequences. The digest was extracted with an equal volume of phenol:chloroform (5:1 [wt/vol]), and the RNA was precipitated by centrifugation after a 40-min incubation at -20 °C in the presence of 75 mm sodium acetate buffer (pH 5.2) and 75% (vol/vol) ethanol. First-strand cDNA was synthesized using Tetro cDNA synthesis kit (Bioline), conducted as described in (52). Primers used to analyze the *raf1* transcripts were raf1RTF and raf1RTR (*SI Appendix*, Table S2).

### Protein Extraction from Syn7942 and Immunoblot Analysis.

Cyanobacterial cultures grown to an OD_750_ of ∼1.0 were harvested by centrifugation (6,000 × *g* for 10 min) and resuspended in TE buffer containing 20 mM Tris⋅HCl pH 8.0, 0.5 mM EDTA, and protease inhibitor mixture (Promega). The cells then were broken by sonication at 4 °C for 3 min (5 s + 25 s break for six cycles). The supernatant obtained after primary centrifugation (5,000 × *g* for 5 min at 4 °C) was further centrifuged (12,000 × *g* for 10 min at 4 °C) to separate the soluble fraction (supernatant) from the insoluble fraction (pellet). Protein concentrations of the soluble fraction were estimated spectrophotometrically at 280 nm, and 20 µg of soluble protein was separated on 10% SDS-PAGE or on 3 to 12% Bis-Tris NativePAGE (Novex). Gels were blotted onto Amersham Hybond PVDF membrane (GE Healthcare) at 90 V for 45 min in a Trans-Blot cell (Bio-Rad). The membrane was immunoprobed using rabbit polyclonal antisera against RbcL (1:10,000; Agrisera) or CcmM (1:1,000; provided by Martin Parry) or CcmK2 (1:1,000; Gemini Biosciences) overnight, followed by goat anti-rabbit HRP-conjugated secondary antibody (1:10,000; Agrisera) for 1 h. Immunoreactive polypeptides were visualized using Clarity Western ECL substrate (Bio-Rad), and signals were captured with an ImageQuant LAS4000 biomolecular imager (GE Healthcare). Signal quantification was performed using Fiji ImageJ. For each experiment, at least three biological repeats were performed.

### Isolation of Carboxysomes and Structural Intermediates.

The isolation method used here was modified based on a previous report (9). Cyanobacterial cells were harvested by centrifugation (6,000 × *g* for 10 min) and resuspended in TE buffer containing 5 mM Tris⋅HCl pH 8.0 and 1 mM EDTA, supplemented with rLysozyme (Merck-Millipore). Cells were incubated at 30 °C in the dark overnight with gentle shaking to enable cell wall degradation, and then collected by centrifugation. Cells were resuspended in ice-cold TE buffer supplemented with Benzonase nuclease (Merck-Millipore), followed by three passages through a high-pressure homogenizer (Stansted) at a homogenizing pressure of 150,000 psi. Triton X-100 (Sigma-Aldrich) was added to a final concentration of 1% (vol/vol), and the broken cells were mixed by inversion on a rotating shaker at 4 °C for 1 h. Cell debris and unbroken cells were removed by centrifugation at 7,000 × *g* for 30 min, and the supernatant was centrifuged at 10,000 × *g* for 10 min 4 °C. The supernatant was further centrifuged at 40,000× g for 30 min 4 °C to generate a crude carboxysome pellet, which was washed and incubated in TEMB buffer (5 mM Tris⋅HCl pH 8.0, 1 mM EDTA, 10 mM MgCl_2_, 20 mM NaHCO_3_) containing 0.5% n-dodecyl-β-maltoside (Sigma-Aldrich) and Benzonase nuclease (Merck-Millipore) for 4 h at 4 °C overnight. The final pellet was resuspended in TEMB buffer and clarified by centrifugation at 6,000 × *g* for 2 min, followed by loading onto a 10 to 60% linear sucrose gradient in TEMB buffer. Gradients were ultracentrifuged at 105,000 × *g* for 1 h, and each gradient was collected and tested by SDS-PAGE and immunoblotting. The gradients at the 20% and 50% sucrose fractions were used for EM analysis.

To verify the destiny of CcmK2, total protein extractions from the WT and Δ*raf1* mutant after cell breakage were first centrifuged at 10,000 × *g* for 10 min at 4 °C to remove unbroken cell debris and insoluble protein aggregates and membrane fragments. The resulting supernatants were then centrifuged at 40,000 × *g* for 30 min at 4 °C to pellet carboxysome structures from the soluble fraction. The resulting supernatant and pellet were analyzed by SDS-PAGE and immunoblotting using an anti-CcmK2 antibody.

### TEM.

Cyanobacterial cell cultures were pelleted by centrifugation (6,000 × *g* for 10 min) and processed for thin sections using a Pelco BioWave Pro laboratory microwave system. The cells were first fixed with 2.5% glutaraldehyde in 0.1 M sodium cacodylate buffer at pH 7.2 using two steps of 100 W. After agarose embedding, the samples were stained with 2% osmium tetroxide and 3% potassium ferrocyanide using three steps of 100 W. The osmium stain was set using 1% thiocarbohydrazide and 2% osmium tetroxide. The samples were stained with 2% uranyl acetate before dehydration by increasing the alcohol concentration from 30% to 100% and increasing resin embedding. Thin (70-nm) sections were cut with a diamond knife and poststained with 3% lead citrate. For isolated carboxysomes, samples were immobilized onto the glow-discharged grids and then stained with 2% uranyl acetate. TEM imaging was conducted using an FEI Tecnai G2 Spirit BioTWIN transmission electron microscope equipped with a Gatan Rio 16 camera.

### Cryo-EM Sample Preparation, Data Collection, and Processing.

Holey carbon-supported copper grids (Quantifoil R2/1; 300 mesh) were plasma-cleaned for 10 s before use. The cryo-EM sample grids were frozen in liquid ethane using the Vitrobot (FEI). A 3.5- μL protein solution (1.2 mg∙mL^−1^) was added to the grid, which was blotted for 4 s with a blot force of 0 in 100% relative humidity at 8 °C. The grids were stored in liquid nitrogen for future use.

The cryo-EM data were collected at 200 kV with a Tecnai F20 microscope (FEI). Movies (40 frames, each 0.125 s; total dose, 25 e/Å^2^) were recorded using a K2 Summi direct electron-counting detector and K2 camera (Gatan) in the superresolution counting mode with a defocus range of 1.5 to 2.5 μm. The data were acquired using the SerialEM progam at a magnification of 29,000×, yielding a pixel size of 1.25 Å. A total of 3320 movie frames were collected, and the beam-induced motion correction was performed by MotionCor2 (53). The defocus value for each micrograph was determined using CtfFind (54). Approximately 3,000 particles were manually picked out by Relion (55), which was used as a template for automatic picking. A total of 730,000 particles were boxed and subjected to several rounds of 2D and 3D classifications with D2 symmetry by RELION. Eventually, 16,022 good particles were selected for the refinement, which obtained the final map at 4.28-Å resolution (*SI Appendix*, Table S1 and Fig. S2), as determined by gold standard Fourier shell correlation using a cutoff of 0.143.

### Model Building and Refinement.

The final map showed clear features of the secondary structural elements, enabling us to fit RbcL_8_ into the map using CHIMERA (56). The remaining continuous density was found to correlate with the Raf1α domains, which were fitted into the map using CHIMERA. Then automatic model refinement was performed by Real_space_refine in Phenix (57), followed by manual refinement in Coot (58) using the crystal structures of Syn7942 Rubisco (PDB ID code 6HBC) and *Anabaena* sp. PCC 7120 RbcL-Raf1 complex (PDB ID code 6KKM). The final model was evaluated by Molprobity (59). The structural figures were prepared using PyMOL (https://pymol.org/2/).

### Confocal Microscopy and Image Analysis.

Fluorescence microscopy was performed on a Zeiss LSM780 confocal microscope with a 63×/1.4 NA oil-immersion objective and laser lines at 405 nm (for CFP), 488 nm (for GFP), and 514 nm (for YFP) using 5 μL of log-phase cells spotted onto 1% agarose pads as described previously (33). Live-cell images were recorded from at least three different cultures. All images were captured with all pixels below saturation. During time-lapse microscopy sample preparation and imaging, cells were continuously illuminated with incandescent light. Images were captured at 2-s intervals for 30 frames. Image analysis was carried out using Fiji. Tracking analysis was performed similarly as described previously with the FIJI Trackmate plugin (19). Spots were recognized with a DoG detector with a median filter and subpixel localization parameters. Velocities were calculated from spot-to-spot displacement directly from all spot output. More than 300 tracks were analyzed from three different images. Automated analysis of the distribution of carboxysomes in vivo was carried out using Image SXM (https://www.liverpool.ac.uk/∼sdb/ImageSXM/) with a custom-programmed plugin (33).

### In Vivo Carbon Fixation Assays.

Cyanobacterial cells were harvested at the exponential phase and then resuspended in Rubisco assay buffer containing 100 mM EPPS (pH 8.0) and 20 mM MgCl_2_ to a final OD_750_ of 4. Radiometric assays were carried out according to a previously described protocol (60) with additional cell permeabilization treatment (61). Cell cultures prepared in assay buffer with the same cell density were incubated with NaH^14^CO_3_ (final concentration 25 mM) at 30 °C for 2 min and then permeabilized by mixed alkyltrimethylammonium bromide (final concentration 0.03% [wt/vol]; Sigma-Aldrich). RuBP (Sigma-Aldrich) was then added at a range of concentrations (0 to 2.0 mM) to initialize the fixation. After 5 min of reaction, formic acid was added to a final concentration of 10% to terminate the reaction. Samples were then dried on heat blocks at 95 °C to remove unfixed NaH^14^CO_3_, and the pellets were resuspended in distilled water in the presence of a scintillation mixture (Ultima Gold XR; Perkin-Elmer). Radioactivity measurements were carried out using a liquid scintillation analyzer (Tri-Carb 2910 TR; PerkinElmer). Raw readings were processed to determine the amount of fixed ^14^C, calibrated by blank cell samples without providing RuBP, and then converted to the total carbon fixation rates. The measured carbon fixation activities were normalized by the total RbcL content detected by immunoblotting. The maximum Rubisco activity and *K*_m(RuBP)_ values were calculated by fitting to the Michaelis–Menten first-order rate equation using GraphPad Prism. For each experiment, at least three biological repeats were prepared. Significance was assessed using a two-tailed *t* test.

### Data Availability.

The final model (PDB ID code 6SMH) and electron microscopy volume map (EMD ID code 10235) have been deposited in the Protein Data Bank and Electron Microscopy Data Bank, respectively.

## Supplementary Material

Supplementary File

Supplementary File
