# Supplementary material for: Rubisco accumulation factor 1 (Raf1) plays essential roles in mediating Rubisco assembly and carboxysome biogenesis
Source: Proc Natl Acad Sci U S A. 2020 Jul 7;117(29):17418–28. doi: 10.1073/pnas.2007990117 (PMC7382273; doi:10.1073/pnas.2007990117)
Supplement: Supplementary File [file pnas.2007990117.sapp.pdf]

PNAS
www.pnas.org

Supplementary Information for

Rubisco accumulation factor 1 (Raf1) plays essential roles in mediating Rubisco assembly and carboxysome biogenesis

Fang Huang[a], Wen-Wen Kong[b,#], Yaqi Sun[a,#], Taiyu Chen[a], Gregory F. Dykes[a], Yong-Liang Jiang[b], Lu-Ning Liu[a,c,*]

[a] Institute of Integrative Biology, University of Liverpool, Liverpool L69 7ZB, United Kingdom

[b] School of Life Sciences, University of Science and Technology of China, Hefei, Anhui 230027, China.

[c] College of Marine Life Sciences, and Frontiers Science Center for Deep Ocean Multispheres and Earth System, Ocean University of China, Qingdao 266003, China

* To whom correspondence may be addressed: Lu-Ning Liu, Luning.Liu@liverpool.ac.uk.

[#] Equal contribution

This PDF file includes:

Figures S1 to S14
Tables S1 to S2
Legend for Movie S1

Other supplementary materials for this manuscript include the following:

Movie S1

www.pnas.org/cgi/doi/10.1073/pnas.2007990117

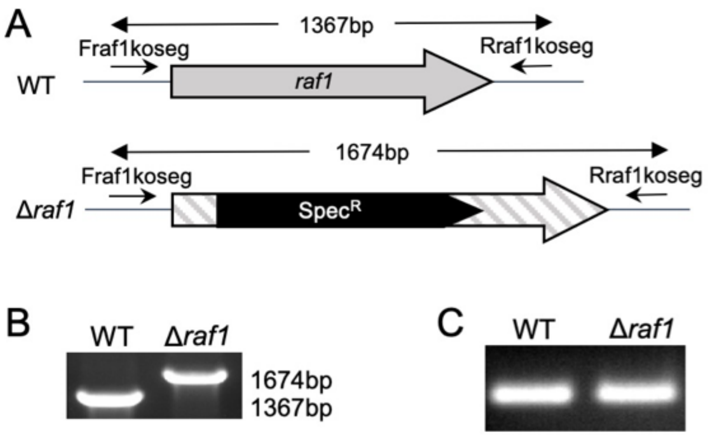

Fig. S1. Generation and verification of the Syn7942 Δ*raf1* mutant. (A) Scheme for insertional inactivation of *raf1*, by replacing the gene with a Spec resistance cassette. Hybridization positions of primers used for full segregation genotyping of the mutation and the size of the expected amplified fragments were indicated. (B) Verification of *raf1* insertional mutation and homogeneity by PCR. (C) Comparison of relative abundance of *rbcL* mRNA in WT and Δ*raf1* mutant by RT-PCR (experiments were repeated three times).

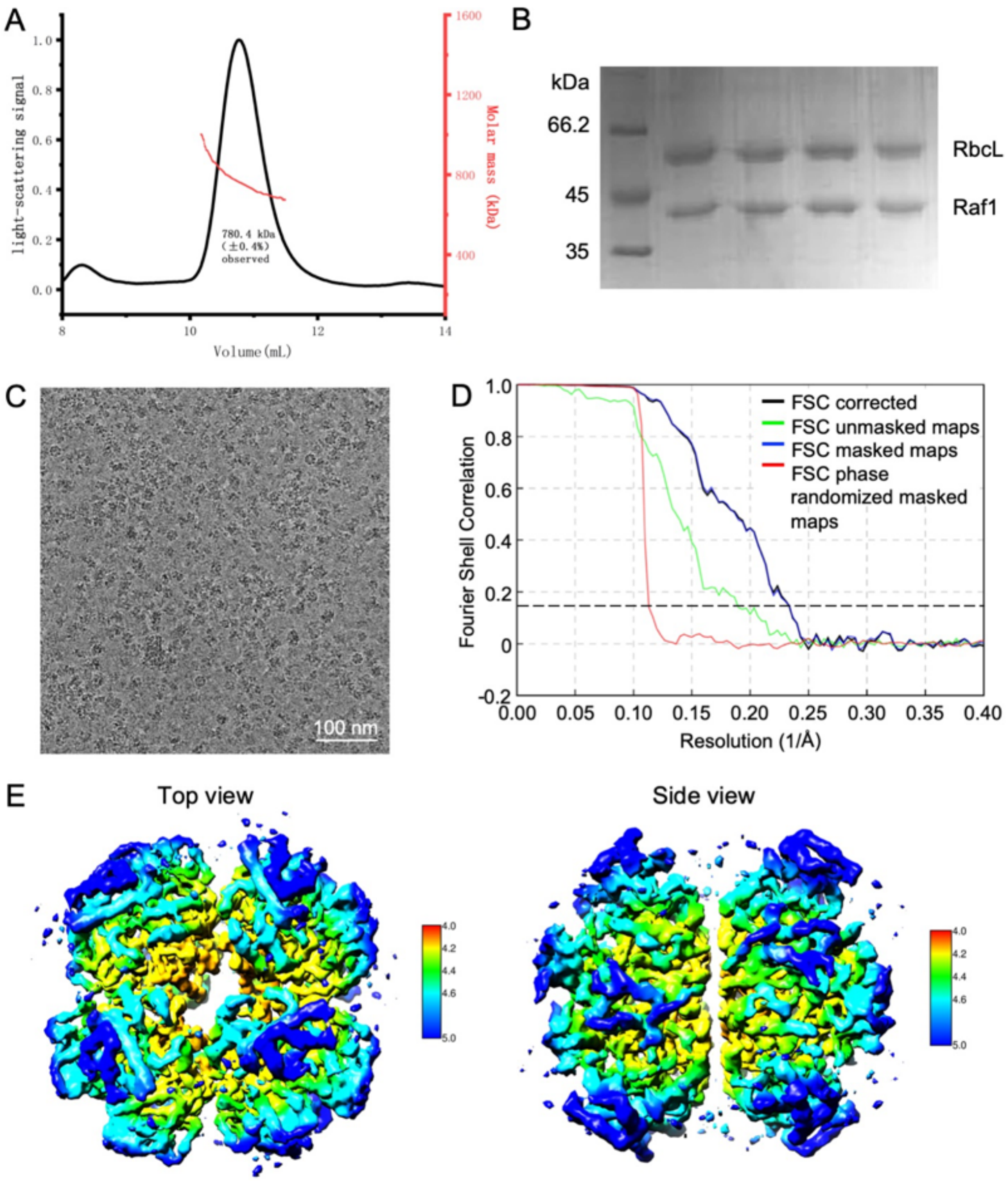

Fig. S2. Structural analysis of the Syn7942 RbcL-Raf1 complex. (A) SEC-MALS analysis of purified Syn7942 RbcL-Raf1 complex. (B) RbcL and Raf1 in RbcL-Raf1 complexes separated on SDS-PAGE followed by Coomassie staining. (C) Micrograph of Syn7942 RbcL-Raf1 complexes. (D) Fourier Shell Correlation (FSC) curves of Syn7942 RbcL-Raf1 complex as determined by gold standard FSC procedure in RELION. (E) Local resolutions of the top-view and side-view cryo-EM map of RbcL-Raf1.

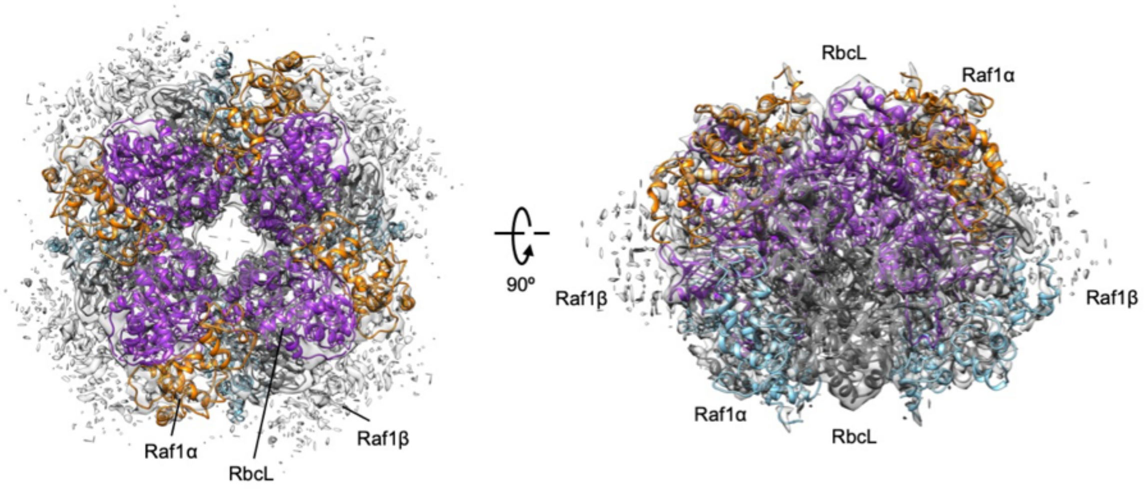

Fig. S3. The top-view (left) and side-view (right) structure of the $RbcL_8$-$Raf1\alpha_8$ complex (PDB: 6SMH) overlapped with the electron density map.

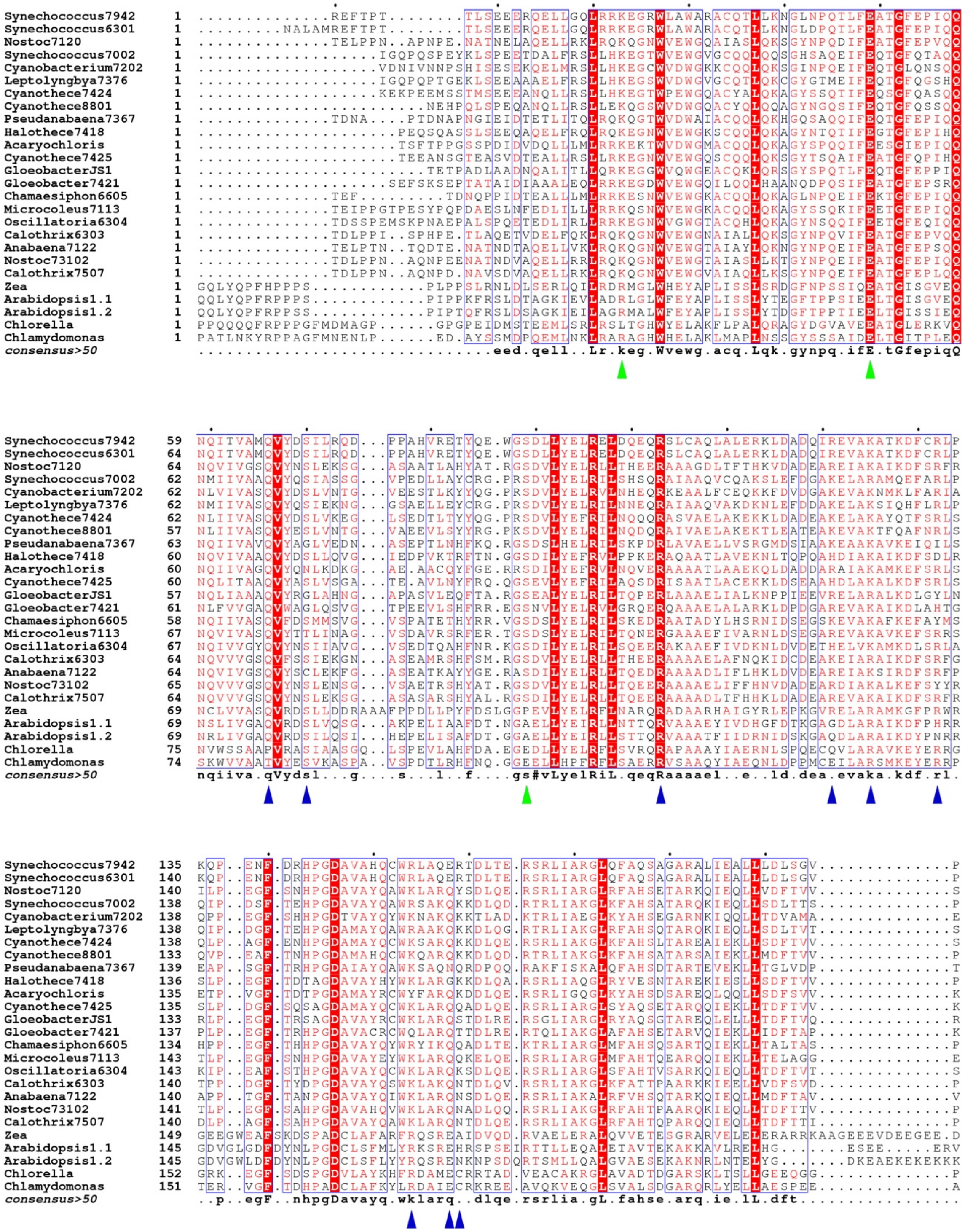

Fig. S4. Alignment of Raf1 α-domain sequences. Amino acid sequences of a set of Raf1 homologs were aligned using ESPript3.0. Identical residues are shown in white on red background. Similar residues are shown in red. Blue frames indicate homologous regions. Blue arrowheads indicate the amino acid residues of Raf1α that interact with RbcL dimer 1. Green arrowheads indicate the amino acid residues of Raf1α that interact with RbcL dimer 2.

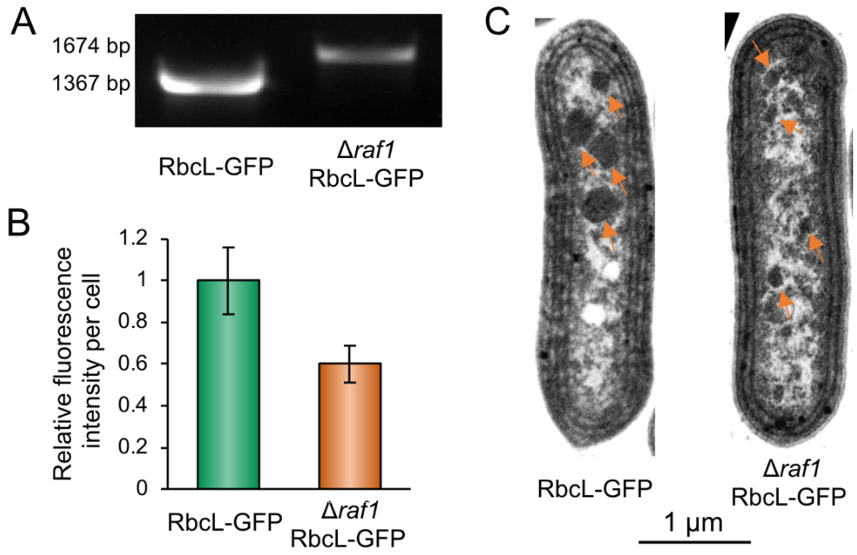

Fig. S5. Characterization of the RbcL-GFP and Δ*raf1*/RbcL-GFP cells. (A) PCR verification of the *raf1* insertional mutation and homogeneity in the Δ*raf1*/RbcL-GFP mutant. (B) Quantification of the average GFP signal per cell in RbcL-GFP and Δ*raf1*/RbcL-GFP (n = 100). (C) Thin-section EM images of RbcL-GFP and Δ*raf1*/RbcL-GFP cells.

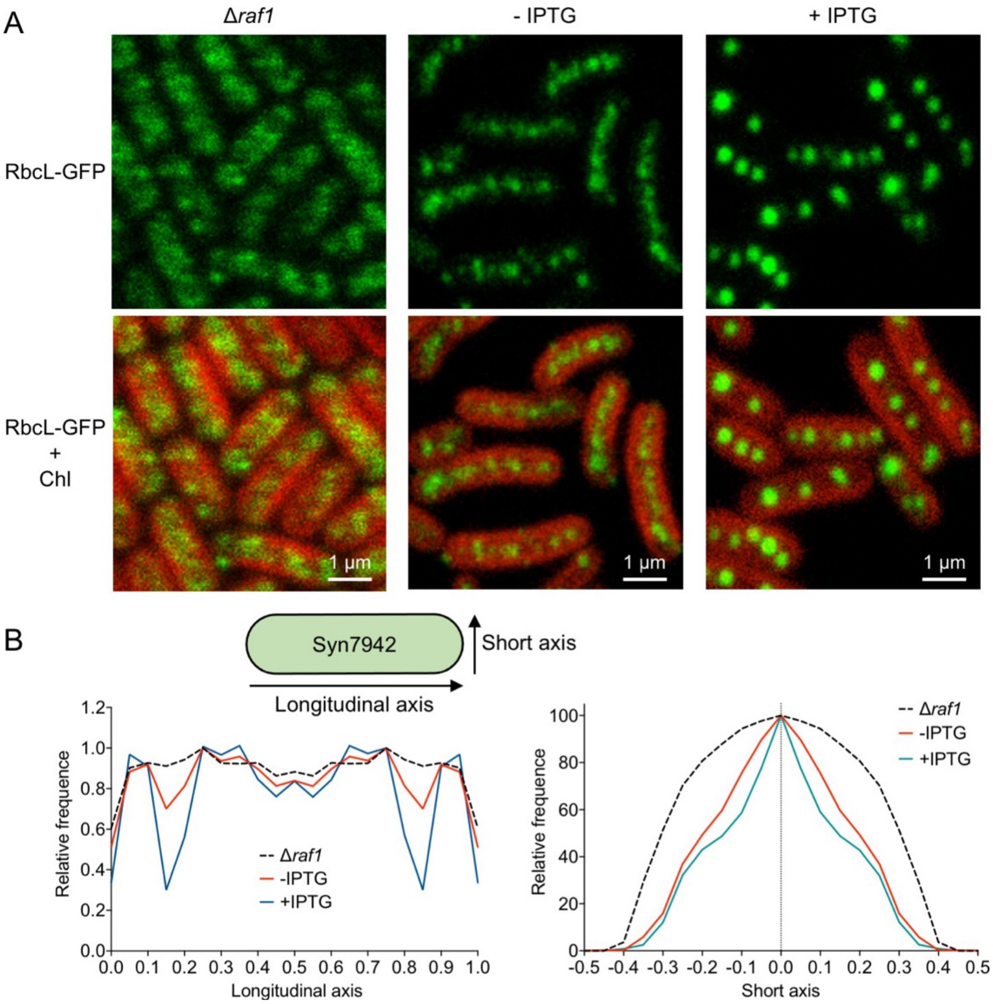

Fig. S6. Complementation of Syn7942 Raf1 induced by IPTG led to the recovery of carboxysome assembly in the Δ*raf1*/RbcL-GFP mutant. (A) Confocal images of the Δ*raf1* cells (control), Δ*raf1* containing the Raf1-expression vector without IPTG induction (-IPTG) and with IPTG induction (+IPTG). Confocal images were recorded from at least three different cultures for each sample. (B) Statistical analysis of the spatial localization of fluorescence signals of the assembly intermediates and carboxysome structures along both the longitudinal and short axes of the cells (*n* = 50) (following the method reported in reference 33 in the main text). IPTG-induced WT Syn7942 Raf1 expression could recover carboxysome formation and typical segmentation in Syn7942. Note that the localization of RbcL-GFP in -IPTG shows an intermediate status between those of Δ*raf1* and +IPTG cells, probably due to the leaky expression of Raf1 under the control of the *lac* promoter. The results indicate the dose-dependent effects of Raf1 on carboxysome formation and localization. During the gradually increase of Raf1 abundance, there might be a range of different assembly intermediates formed in the Δ*raf1* cells.

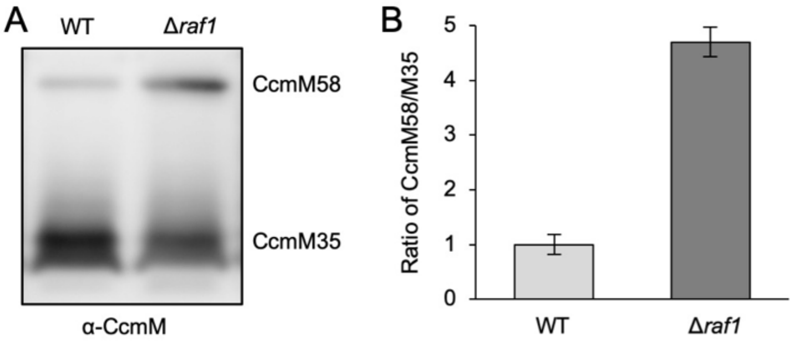

Fig. S7. The stoichiometric ratio of long-term and short-term CcmM (CcmM58/CcmM35) in the WT and Δ*raf1* mutant. (A) Immunoblot analysis using an anti-CcmM antibody shows distinct ratios of CcmM58 and CcmM35 in the WT and Δ*raf1* mutant. (B) Quantification of the ratios of CcmM58 and CcmM35 based on immunoblot results from three independent experiments.

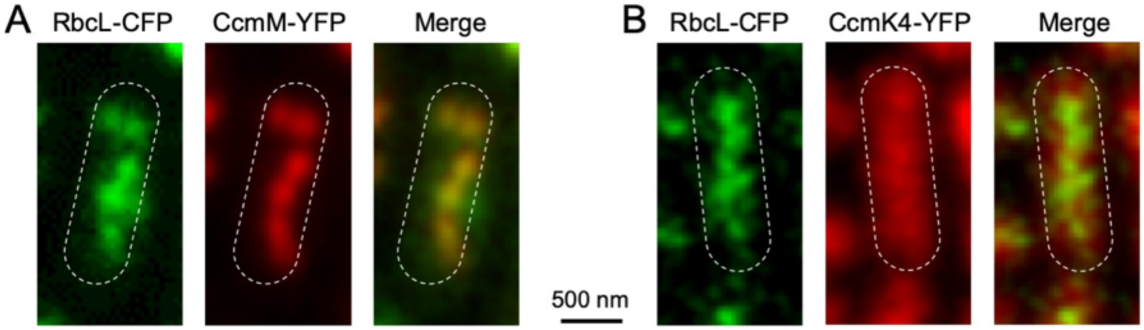

Fig. S8. Colocalization analysis of RbcL and CcmM as well as RbcL and CcmK4 in the Δ*raf1* mutant. (A) Confocal images of the Δ*raf1*/RbcL-CFP/CcmM-YFP cells reveal the colocalization of Rubisco and CcmM. Confocal images were recorded from three different cultures. (B) Confocal images of the Δ*raf1*/RbcL-CFP/CcmK4-YFP cells indicate that Rubisco and CcmK4 do not colocalize *in vivo*. Confocal images were recorded from three different cultures.

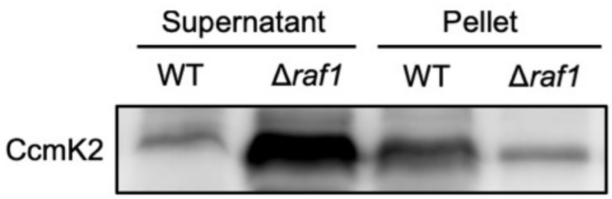

Fig. S9. CcmK2 content in the supernatants and pellets of WT and Δ*raf1*. Immunoblot analysis using an anti-CcmK2 antibody shows a large amount of CcmK2 proteins in the supernatant of the Δ*raf1* mutant, in contrast to the fact that the majority of CcmK2 proteins in the WT were detected in the carboxysome-enriched pellet after a 40,000× g centrifugation. The results revealed that the major shell proteins CcmK2 were not included in the resulting structural intermediates.

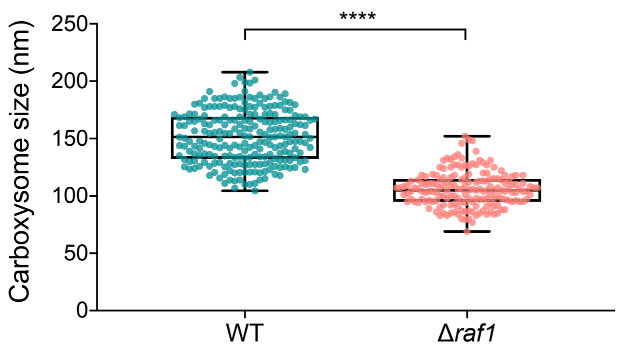

Fig. S10. Comparison of the size of carboxysome structures isolated from the WT and Δ*raf* cells, determined by EM. WT carboxysomes: 151.6 ± 23.1 nm in diameter (n = 234); Δ*raf* carboxysomes: 105.9 ± 15.18 nm in diameter (n = 180). The diameter measurement was performed as previously described (see reference 9 in the main text). ****, $p < 0.0001$.

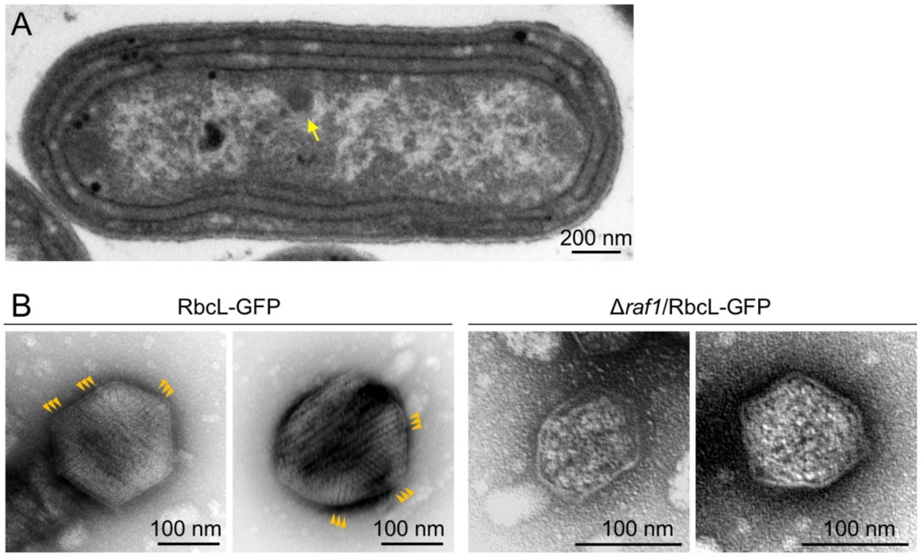

Fig. S11. EM characterization of carboxysome-like structures. (A) Thin-section EM of the Δ*raf* cells reveals occasionally some small carboxysome-like structures (arrow). (B) EM images of isolated carboxysome(-like) structures captured at the 50% sucrose fractions of RbcL-GFP (left) and Δ*raf*/RbcL-GFP cells (right). Consistent with the non-GFP fused carboxysome particles (Fig. 5), RbcL-GFP carboxysome structures are ~150 nm in diameter and exhibit regular polyhedral shell shapes (straight edges) and densely packed interior arrays (arrows), whereas Δ*raf*/RbcL-GFP carboxysome-like structures possess a smaller size (<100 nm in diameter), irregular polyhedral shell shape (curved edges), and disordered interior packing.

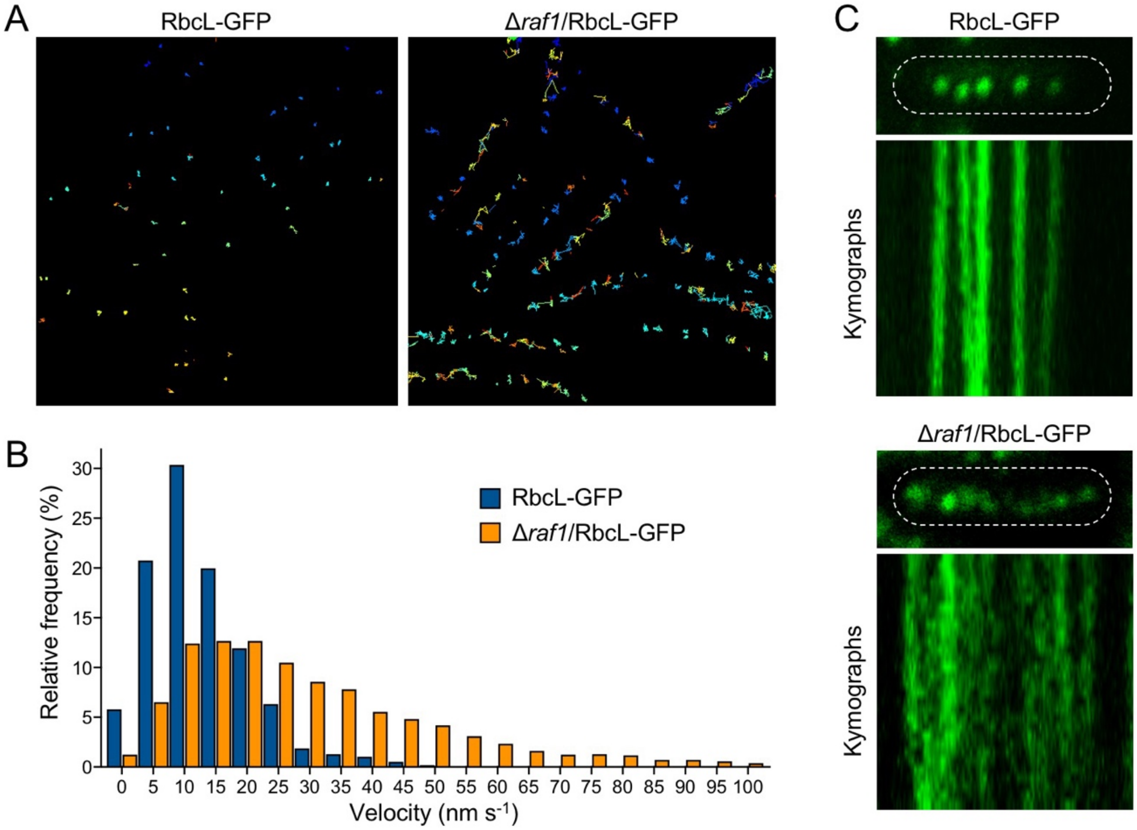

Fig. S12. Diffusion dynamics of carboxysomes and assembly intermediates in RbcL-GFP and Δ*raf*/RbcL-GFP cells. (A) Particle tracking overlaid on one frame during the 60 s tracking with the time interval of 2 sec. See also Movie S1. Different tracks are illustrated in different colors. (B) Histogram of the mean velocities of tracked carboxysomes and assembly intermediates in the RbcL-GFP and Δ*raf*/RbcL-GFP mutants. The velocities of carboxysomes in RbcL-GFP cells and assembly intermediates in Δ*raf*/RbcL-GFP cells are 12.6 $nm \cdot s^{-1}$ (n = 1500) and 29.3 $nm \cdot s^{-1}$ (n = 2600), respectively. (C) Kymographs of RbcL-GFP signals over the longitudinal axis of RbcL-GFP and Δ*raf*/RbcL-GFP cells in the 60 s tracking, revealing the greater oscillation of assembly intermediates in Δ*raf*/RbcL-GFP cells than that of carboxysomes in RbcL-GFP cells.

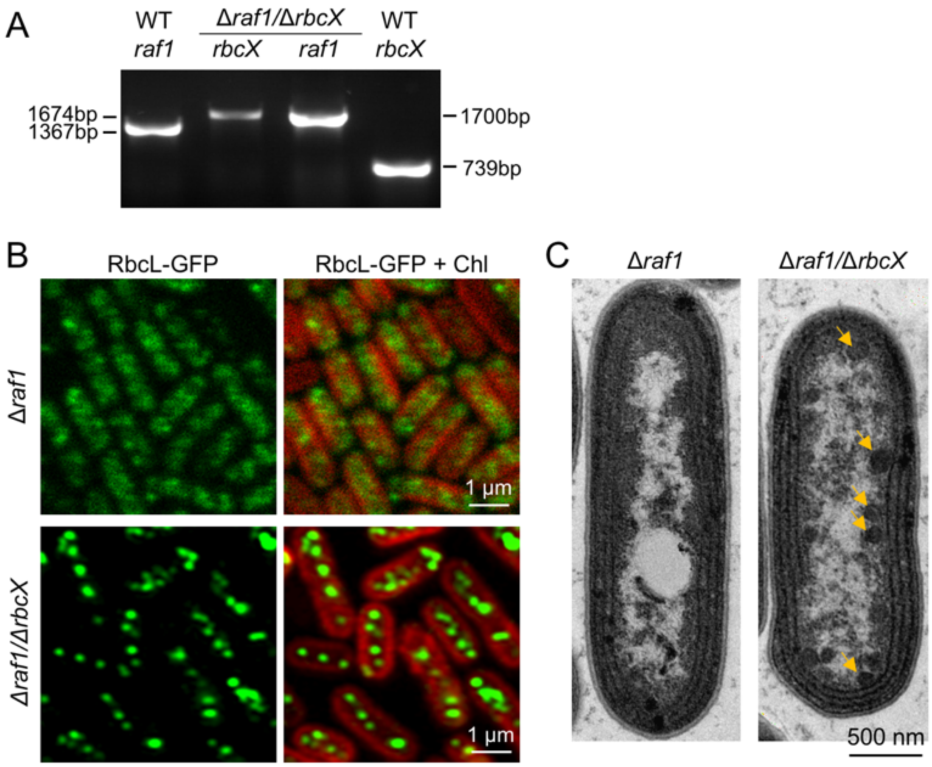

Fig. S13. Functional correlation between Raf1 and RbcX. (A) PCR verification of the *raf1* and *rbcX* insertional mutation and homogeneity in the Δ*raf1*/Δ*rbcX*/RbcL-GFP mutant. (B) Confocal images of the Δ*raf1*/RbcL-GFP and Δ*raf1*/Δ*rbcX*/RbcL-GFP cells show the deletion of *rbcX* in the Δ*raf1* mutant could induce partially carboxysome formation. Confocal images were recorded from three different cultures. (C) Thin-section EM images reveal the occurrence of electron-dense structures induced by *rbcX* deletion of in Δ*raf1* mutant cells.

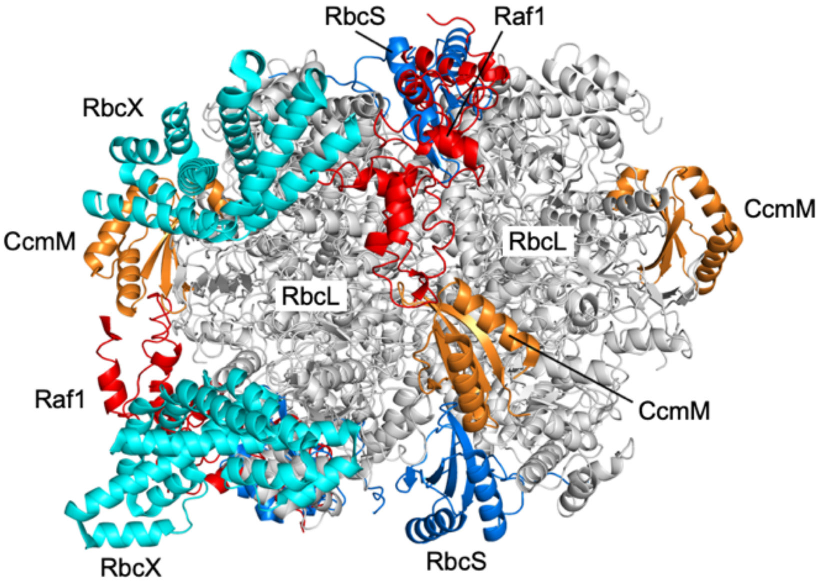

Fig. S14. Superimposed structure of the Rubisco-chaperone supercomplexes comprising $RbcL_8$-$Raf1_8$ (PDB: 6SMH, this study), RbcS (PDB: 6HBC), CcmM (PDB: 6HBC), and RbcX (PDB: 2WVW).

Table S1. Cryo-EM data collection, structural refinement, and validation statistics

	**RbcL-Raf1^{7942}** (EMDB: 10235) (PDB: 6SMH)
**Data collection and processing**	
Magnification	29,000
Voltage (kV)	200
Electron exposure (e^{-}/Å^2)	25
Defocus range (μm)	-1.5~-2.5
Pixel size (Å)	1.25
Symmetry imposed	D2
Initial particle images (no.)	730,000
Final particle images (no.)	16,022
Map resolution (Å)	4.28
FSC threshold	0.143
Map resolution range (Å)	2.50-999
**Refinement**	
Initial model used (PDB code)	6KKM
Model resolution (Å)	4.3
FSC threshold	0.143
Map sharpening *B* factor (Å^2)	-231.74
Model composition	
Non-hydrogen atoms	40,034
Protein residues	5,026
Ligands	0
B factors (Å^2)	106.37
R.M.S. deviations	
Bond lengths (Å)	0.007
Bond angles (°)	1.199
Validation	
MolProbity score	3.72
Clashscore	52.41
Poor rotamers (%)	14.46
Ramachandran plot	
Favored (%)	89.58
Allowed (%)	10.10
Disallowed (%)	0.32

Table S2. Primers used in this work.

Primers	Sequence (5'-3')
raf1F	GCTGAATCCTTGGCTCAACC
raf1R	CGCCCAAGTTAATCCGAGC
raf1koF	TGATATTTATCCGTCTTGTTTGTGAATGCTCTTGCGATGATTCCGGGAT CCGTCGACC
raf1koR	TTGGCAGCGATCGCAGGGTTGAGGAGCGCGGGGCATCTATGTAGGCTG GAGCTGCTTC
raf1domainkoF	CTGCTAGATCTCAGCGGGGTGCCCAGTCGCAAACCAATTCCGGGGATCC GTCGACC
raf1kosegF	CGAAGAACTGCCGAGCATC
raf1kosegR	CCATAGCGGTAGTGCGGATT
raf1RTF	GGACAACAGTGGCTGGCATT
raf1RTR	GGGCGACCACTGAATCCTTG
GroES/EL_F	TACCATGGGCAGCAGCATGGCAGCTGTATCTCTGAG
GroES/EL_R	AATTCGGATCCTGGCTTTAGTAGTCGAAGTCGCCCA
p19RbcLF	CGACGACGACAAGCATATGCCCAAGACGCAATCTGC
p19RbcLR	CAGCCGGATCCTCGAGTTAGAGCTTGTCCATCGTTT
p19Raf1F	TCTAAAAGAAGGAGATATACCATGCGTGAGTTCACCCCCACAACT
p19Raf1R	CAGCCGGATCCTCGAGCTATTCTTCAAACTGCCAAG
pAMraf1F	GAATTCTGAATGCTCTTGCGATGCGT
pAMraf1R	GGATCCGCGGGGCATCTATTCTTCAA

Movie S1 (separate file). Diffusion dynamics of carboxysomes and assembly intermediates in WT and Δ*raf1* cells (2 sec time intervals, 30 frames). Analysis of the diffusion dynamics is shown in Fig. S11.
